# Repigmentation of vitiligo with 5-fluorouracil tattooing in combination with topical ruxolitinib: A case report

**DOI:** 10.1016/j.jdcr.2025.01.016

**Published:** 2025-02-05

**Authors:** Betul Macit, Laura Burns, Carlos Gustavo Wambier

**Affiliations:** Department of Dermatology, The Warren Alpert Medical School of Brown University, Providence, Rhode Island

**Keywords:** 5-fluorouracil, 5-FU, dermal drug-delivery, drug tattooing, melanocytes, repigmentation, ruxolitinib, vitiligo

## Introduction

Vitiligo is a chronic autoimmune disorder that causes skin depigmentation, significantly affecting patients’ quality of life. 5-Fluorouracil (5-FU) has been explored as an alternative treatment for vitiligo.^1^^,^^2^ Its exact mechanisms for inducing hyperpigmentation and repigmentation remain to be fully understood; 5-FU induces DNA damage, apoptosis, and chemotaxis of melanocytes through endothelin-1, ACTH, ɑ-MSH, cAMP, cytokines, prostaglandins, and increased expression of tyrosinase.^3^

Because of its highly polar nature, 5-FU has low cell membrane affinity when applied topically, limiting its penetration into deeper skin layers. Thus, specialized delivery methods are required to bypass the corneal layer barrier and reach the epidermis.^4^ Various techniques, including microinfusion (drug tattooing), dermabrasion, ablative laser, microneedling, and intradermal injection, have been explored to enhance penetration.^5^

Building on the success of 5-FU tattooing in treating idiopathic guttate hypomelanosis, we have routinely applied this innovative approach to vitiligo.^6^ The technique involves delivering 5-FU into the papillary dermis using a professional tattoo machine, effectively reaching the deeper layers of the skin. By creating a favorable microenvironment in the papillary dermis, 5-FU tattooing may promote long-lasting melanocyte migration and pigment spread in vitiligo, offering a novel and potentially more effective treatment strategy.^7^

## Case report

A 52-year-old woman presented to our dermatology clinic with skin discoloration in the bilateral axillae. Eight months earlier, she noticed pink scaly papules with pruritus that gradually led to decreased skin pigmentation. She was initially treated with pimecrolimus cream without improvement. Her medical history included alopecia areata but no other autoimmune disorders.

**Fig 1:**
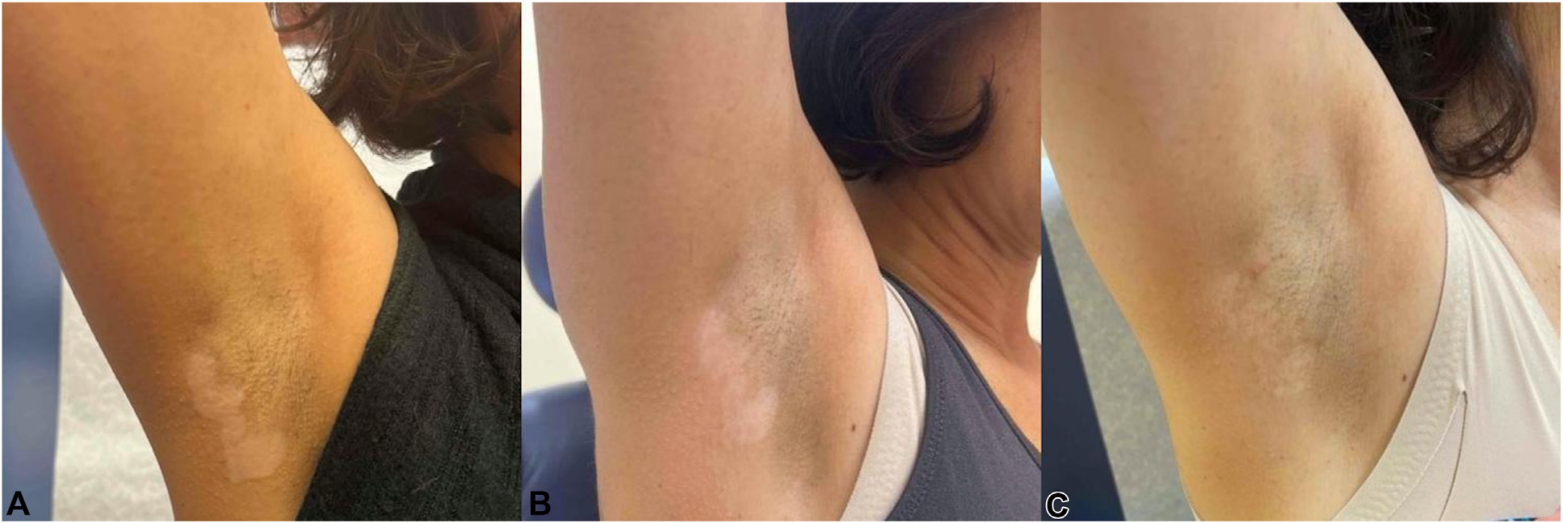
Vitiligo. Right axilla progression of treatment. **A,** Depigmented patches consistent with vitiligo before treatment. **B,** Slight reduction in the depigmented area after 7 months of ruxolitinib 1.5% cream twice daily as monotherapy before tattooing. **C,** Partial repigmentation observed 4 months after a single session of 5-fluorouracil tattooing, with continuation of ruxolitinib 1.5% cream twice daily.

**Fig 2:**
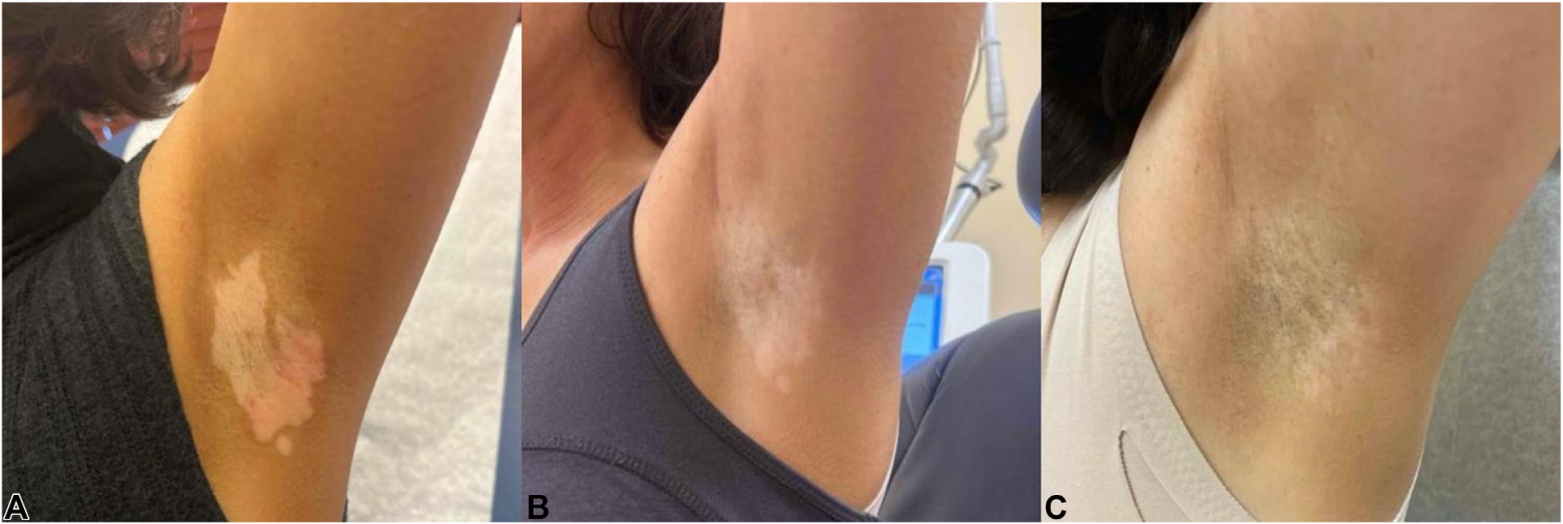
Vitiligo. Left axilla progression of treatment. **A,** Depigmented patches consistent with vitiligo before treatment. **B,** Slight reduction in the depigmented area after 7 months of ruxolitinib 1.5% cream twice daily as monotherapy before tattooing. **C,** Partial repigmentation observed 4 months after a single session of 5-fluorouracil tattooing, with continuation of ruxolitinib 1.5% cream twice daily.

A follow-up evaluation, including Wood’s lamp examination, revealed fluorescence in the depigmented areas. Additionally, depigmented patches were observed around her eyes, nose, and mouth. A clinical diagnosis of vitiligo was strongly favored at this point. Consequently, the patient was started on topical ruxolitinib 1.5% cream, applied twice daily (Figs 1, *A* and 2, *A*).

After 7 months of ruxolitinib, partial improvement was observed in the left axilla, with the depigmented area reducing by 24.3%. The right axilla showed partial improvement, with a 36.5% reduction in depigmented area (Figs 1, *B* and 2, *B*). These reductions were measured by a single evaluator (BM) using ImageJ (NIH). Despite these reductions, the patient reported no significant visible improvement in either area and was agreeable to attempt a drug-delivery medical procedure.

Drug tattooing was performed with 5-fluorouracil (5-FU) 5% sterile injectable solution (Fresenius Kabi USA, LLC) to the depigmented areas, using a Cheyenne SOL Nova Unlimited tattoo machine with 3.5 mm stroke (amplitude). The machine was set at 100 Hz at responsive mode, 1.5 mm throw (needle exposure length) of a 15-Magnum soft-edge capillary safety cartridge with 0.35 texturized needles (MT, Derm GmbH). Patient was advised to continue topical ruxolitinib in the next day.

At her follow-up, 4 months postprocedure, significant repigmentation was observed. In the right axilla, the depigmented area was further reduced by 57.9% from the initial measurement. In the left axilla, the depigmented area decreased by 70.9% from the initial measurement (Figs 1, *C* and 2, *C*). The patient experienced no adverse effects and expressed satisfaction with the results, deciding against further treatment. The area was not subject to sun exposure or UV therapy after the procedure, because the patient had failed radiation exposure before (Figs 1, *A* and 2, *A*).

## Discussion

Our case report presents a novel approach using 5-FU delivered via tattooing in combination with topical ruxolitinib. The use of a professional tattoo machine to deliver 5-FU into the papillary dermis is a key innovation, as it likely overcomes the drug’s hydrophilic nature, which typically limits its ability to penetrate the corneal layer effectively.

The mechanism by which 5-FU induces repigmentation in vitiligo remains unclear. It is speculated that the necrosis/apoptosis of keratinocytes may provide melanocytes with a conducive environment for proliferation, migration, and pigment production.^1^ Similar sequences of events are also known to occur with DNA damage caused by UV-A or UV-B radiation.^8^ The combination of Janus kinase inhibitors with narrow-band UV-B or sun exposure has been subject to extensive research over the past years.^9^

Previous studies have explored various combination therapies involving 5-FU, such as intradermal injections, dermabrasion, erbium laser ablation, and microneedling, with results ranging from moderate to excellent repigmentation.^6^^,^^7^ Among these, microneedling with 5-FU has been one of the more extensively studied approaches. However, the traditional microneedling technique, which involves the creation of microinjury columns in the skin, does not always facilitate optimal dermal delivery of therapeutic agents. Pressure gradients can limit the penetration of therapeutic agents, in microneedling with dry needles followed by topical application, the bleeding flow to the epidermis and fibrin does not allow liquid to penetrate in the injury columns. The tattooing technique (wet needles), in contrast, generates negative pressure from needle withdraw that delivers fluids surrounding the needle into the papillary dermis. Traditionally developed for depositing color pigments, this technique has been adapted to achieve more uniform and effective delivery of 5-FU and other drugs, potentially improving outcomes not only in vitiligo but also in other dermatologic conditions where targeted dermal delivery is crucial.^10^

Although this single-patient case is promising, limitations must be acknowledged. Further research with larger cohorts is necessary to validate these findings and explore the broader applicability of 5-FU tattooing in vitiligo treatment.

Future studies could also investigate the long-term effects and potential for combining 5-FU tattooing with other therapeutic modalities. Although the combined use of 5-FU and topical ruxolitinib demonstrated significant repigmentation in this case, there is no current literature supporting a mechanistic interaction between these treatments. Future studies could recruit patients with axillary vitiligo (which take away any confounding from ambient UV light), use split-treatment designs, and perform biopsies at 4 to 6 months posttreatment to analyze cellular markers and skin pathology. Such studies would help to further elucidate the mechanisms underlying 5-FUs effects on melanocyte activity and repigmentation.

## Conflicts of interest

Dr Wambier has served as consultant/adviser for Incyte, Chemistry Rx, Vydence, and Young Pharmaceuticals, as a speaker for Pfizer, and as an investigator for Incyte, Sun Pharma, Eli Lilly, and Pfizer. The other authors have no conflicts of interest to declare.
